# Deep Learning for Detecting Multi-Level Driver Fatigue Using Physiological Signals: A Comprehensive Approach

**DOI:** 10.3390/s23198171

**Published:** 2023-09-29

**Authors:** Mohammad Peivandi, Sevda Zafarmandi Ardabili, Sobhan Sheykhivand, Sebelan Danishvar

**Affiliations:** 1 Department of Biomedical Engineering, Wayne State University, Detroit, MI 48202, USA; hm95599@wayne.edu; 2 Electrical and Computer Engineering Department, Southern Methodist University, Dallas, TX 75205, USA; szafarmandiardabilii@smu.edu; 3 Department of Biomedical Engineering, University of Bonab, Bonab 55517-61167, Iran; s.sheykhivand@tabrizu.ac.ir; 4 College of Engineering, Design and Physical Sciences, Brunel University London, Uxbridge UB8 3PH, UK

**Keywords:** deep learning, feature extraction, GAN, CNN, EEG, physiological signals, machine learning, driver fatigue

## Abstract

A large share of traffic accidents is related to driver fatigue. In recent years, many studies have been organized in order to diagnose and warn drivers. In this research, a new approach was presented in order to detect multi-level driver fatigue. A multi-level driver tiredness diagnostic database based on physiological signals including ECG, EEG, EMG, and respiratory effort was developed for this aim. The EEG signal was used for processing and other recorded signals were used to confirm the driver’s fatigue so that fatigue was not confirmed based on self-report questionnaires. A customized architecture based on adversarial generative networks and convolutional neural networks (end-to-end) was utilized to select/extract features and classify different levels of fatigue. In the customized architecture, with the objective of eliminating uncertainty, type 2 fuzzy sets were used instead of activation functions such as Relu and Leaky Relu, and the performance of each was investigated. The final accuracy obtained in the three scenarios considered, two-level, three-level, and five-level, were 96.8%, 95.1%, and 89.1%, respectively. Given the suggested model’s optimal performance, which can identify five various levels of driver fatigue with high accuracy, it can be employed in practical applications of driver fatigue to warn drivers.

## 1. Introduction

One of the leading causes of traffic accidents is driver weariness [1]. It is estimated that about 40% of road accidents happen due to the sleepiness and fatigue of drivers [2]. According to the report provided by the United States Traffic Safety Administration, 350,000 traffic accidents occur due to driver fatigue every year, of which 11,000 accidents result in injuries and 70,000 accidents result in the deaths of drivers and passengers. Based on the mentioned cases, timely diagnosis of driver fatigue and warning drivers is of great importance and has received a lot of attention in recent years [1,2].

There are two categories of exhaustion: physical fatigue and mental fatigue. Physical weariness results from not getting enough sleep and performing duties. However, undertaking difficult activities, like driving, might cause mental tiredness. In the past, the neuroimaging method of electroencephalogram (EEG) has been most frequently used to study mental weariness. It has been well established that mental weariness may cause clear modifications in EEG readings. Theta power and alpha power have substantial positive and negative relationships, respectively, with subjective tiredness in the resting state of the EEG. The strength of the alpha rhythm increases when the eyes are open and diminishes when the eyes are closed as mental tiredness increases. It is possible to identify mental exhaustion using these changes in the EEG, which is crucial and significant for estimating driving fatigue [3,4].

In general, three basic methods are used for driver fatigue detection: monitoring the facial condition and facial expression based on processing algorithms, detection based on the road markings and the angle of the car, and physiological monitoring of the driver’s condition. In the first case, driver fatigue based on facial expression, blinking, and yawning rhythm of drivers is achieved by installing a camera in the car based on image processing techniques. A lot of research has been conducted in this regard. However, there are problems with this. This type of tracking will be incorrect if the driver is wearing sunglasses. [5,6].

The second method of detecting driver fatigue is based on placing sensors in the steering wheel of the car and installing cameras in the four corners of the car. Sensors and cameras in this type of monitoring warn drivers if they deviate from the road and leave the line. Driver fatigue monitoring based on this method in snowy weather will be accompanied by errors [7,8].

A third technique identifies driver fatigue by detecting physiological signals such as EEGs, electrocardiograms (ECGs), electrooculograms (EOGs), and so on. Among the three investigated methods, it has been proven that physiological monitoring of drivers can be the most effective way to detect driver fatigue. Among the physiological measurements, EEG is the most effective physiological indicator for diagnosing driver fatigue. Because brain activity changes before the driver enters the fatigue phase, it is possible to identify the driver’s fatigue by changing the pattern of brain activities and informing the driver before accidents occur. However, there are also problems in this regard: the number of channels of EEG signals is high and this factor can be uncomfortable for the driver.

As is obvious, many studies have been undertaken in recent years to identify driver fatigue automatically using EEG signals. However, these past studies also possess some major shortcomings, which are mentioned below. The biggest and most critical problem in previous studies has been the binary classification of fatigue and normal state. As it turns out, much research has attained binary classification accuracy of greater than 99%. However, considering the binary state between fatigue and normal, even with a classification accuracy of above 99%, the current findings cannot be translated into practice. As a result, the number of modes examined for driver fatigue should be increased from binary classifications to multiple classes. This second problem can be seen in the identification of the stage between fatigue and normal. All prior studies, when gathering their database based on brain signals, have been satisfied simply with the findings of the questionnaire from the participants. The third difficulty identified in earlier studies is the lack of a comprehensive database for autonomous driver fatigue identification. All of the benchmark databases are associated with two-level automatic driver fatigue identification, which cannot be relied on in real-world evaluations. To improve on prior research, this study collected a complete benchmark database on diverse levels of weariness for the first time among previous studies. Fatigue detection using the suggested database is based not only on self-reports from participants but also changing patterns of physiological signals. Then, after pre-processing, the data were entered into a customized architecture based on convolutional networks (end-to-end) with a type 2 fuzzy activator, and after feature selection/extraction, the samples were divided into five classes: Normal stage, Onset of Fatigue, Mid Fatigue, Warning Of Fatigue, and Complete Fatigue.

The contributions of this study are as follows:

- Providing a comprehensive five-level database in order to automatically detect driver fatigue.
- Confirmation of driver fatigue based on visual confirmation of changing patterns of physiological signals.
- Presenting a customized architecture based on the generative adversarial networks and fusion of convolutional neural network and type 2 fuzzy set (End-to-End).
- The suggested algorithm’s robustness in the presence of a broad variety of external noises.

The rest of the article is organized as follows: Section 2 examines previous research in order to diagnose driver fatigue along with the related advantages and disadvantages. The method of recording physiological signals in order to collect the proposed database is discussed in Section 3. In addition, the algorithms employed in this study are presented in this part. Section 4 details how to pre-process the recorded data as well as the recommended network design. Section 5 discusses the findings of the study and compares them to earlier research. Section 6 is concerned with the conclusion.

## 2. Related Works

In this regard, several studies have been conducted recently, which will be examined further [9,10].

Lee et al. [11] used the combination of wavelet transform (WT) and deep convolutional networks to automatically detect driver fatigue using EEG signals. These researchers first applied the WT to the recorded EEG signals and applied the output result to their proposed network. The classification accuracy based on the proposed architecture of these researchers has reached 83%. Luo et al. [12] used single-channel EEG to automatically detect driver fatigue. In their study, these researchers used adaptive multiphase entropy features and classified the extracted features using common classifications. The final accuracy of the classification by these researchers based on two channels, FP1 and FP2, was reported as 95%. The high classification accuracy and usage of a single channel can be regarded as benefits of this study, whereas the inclusion of classical characteristics can be considered a downside. Sheykhivand et al. [13] identified driver fatigue by using EEG signals, taking into account different areas of the brain. These researchers used deep convolutional networks in their study. The classification accuracy for the automatic detection of the two different states of fatigue and normal was reported to be around 99%. The high accuracy of 99% can be considered an advantage of this research. The inability to identify multi-level fatigue is also a shortcoming of this research. Zeng et al. [14] attempted to detect driver weariness based on brain signals using a limited database of six subjects. For feature analysis and extraction, these researchers employed conventional machine learning methods such as power spectral density (PSD) and empirical mode decomposition (EMD) in their research. In addition, they used the support vector machine (SVM) classifier in this research to differentiate between two groups: fatigued and normal. Their ultimate accuracy was believed to be approximately 94%. Sheykhivand et al. [15] used a combination of compressed sensing (CS) theory and deep convolutional networks to detect driver fatigue in two states, normal and fatigue, using distinct brain regions. In this study, they reached a 99% accuracy in distinguishing between two types of weariness. One advantage of this study was signal compression, which might be useful in applications that operate in real time. The usage of a single-channel EEG signal was also seen to be the second advantage of this study. The primary problem of this study was computational complexity. Subasi et al. [16] employed a flexible WT to identify driver fatigue utilizing an EEG channel. These researchers correctly distinguished two separate stages of fatigue and rest with 97% accuracy. The benefits of this study include accuracy levels above 95%. However, the usage of the classical approach, which creates computational complexity in the algorithm, might be viewed as a downside of this study. Wang et al. [17] used EEG readings from the gated linear unit (GLU) to determine the driver’s weariness. They retrieved parameters such as power ratio, approximation entropy, and mutual information from EEG signals with 87% accuracy in their research. The key limitation of this study is the low categorization accuracy. Zheng et al. [18] employed the PSD of five frequency bands of the EEG signal, including delta, theta, alpha, beta, and gamma, to identify driver exhaustion in two states: fatigue and normal. In this study, they employed EMD analysis to extract features from EEG data and a random forest (RF) classifier to classify feature vectors. In addition, in the study, they employed several machine learning categories to compare with their method and produced a speech of over 97%. The use of numerous EEG channels might be regarded as one of the study’s shortcomings, despite the fact that it has a high classification accuracy. Chen et al. [19] identified the driver’s fatigue from the phase delay index and fed it to a convolutional network based on EEG signals. In their proposed architecture, convolutional layers, a rectifier linear activation unit, an integration layer, and a fully connected layer were used. The final accuracy, precision, and sensitivity of the classification based on their proposed model were around 95%, 95%, and 93%, respectively. The high categorization accuracy was one of the research’s benefits, while the high number of EEG channels used can be considered a limitation of this research. Shahbakhti et al. [20] used a single-channel EEG signal to detect driver weariness. They incorporated traditional methods in their architecture, such as WT and Adaboost classifier. The EEG signal was initially subjected to the WT in their method. The Adaboost classifier was then used to extract linear and nonlinear characteristics from the signal. The maximum categorization accuracy based on the method given by these researchers is claimed to be 90%. The single-channel nature of EEG signals is one of the advantages of this research, while computing complexity is one of the limitations.

## 3. Materials and Procedures

This section is divided into the following subsections. The approach for gathering a multi-level database based on physiological signals is provided in the first subsection. The methods utilized in this study to augment data, select/extract features, and categorize different levels of fatigue area described in the second portion.

### 3.1. Database Configuration

The specifics of the recorded database, as well as the signal registration possibilities, will be detailed in this part. For this purpose, the necessary permits were obtained from the ethics committee of Tabriz University (license number IR.Tabrizu.1399) and written consent from 20 students (undergraduate, master’s, and doctoral) who had a driver’s license was provided. The participants, who were between the ages of 19 and 40, were asked to participate in the driver fatigue monitoring experiment using a simulator.

The driving simulator was built manually in the signal processing laboratory of Tabriz University. The steering wheel, gearbox, and pedals of Logitech G29 company (Suzhou, China) were used for the proposed simulator. A 42-inch Samsung TV (Samsung Electronics, Suwon, South Korea) was used to show the car’s windshield to the driver. Also, a profile holder for installing the steering wheel, gear box, and pedals was designed and then made using Corel software. In addition, a Peugeot 405 passenger car seat (Peugeot, Citroën and Renault, French) was used in order to simulate real driving for the driver by installing it on a stand to adjust the height. The chair used for the simulator was able to have its back adjusted in several modes to adjust for subjects with different heights and weights. City Car Driving version 1.5 simulation software was used to simulate the driving environment by changing the software source for personalization. A partition wall was used to simulate the car cabin. A separating wall was used to prevent subjects from being distracted while driving.

All participants were free of neurological and mental diseases such as depression, epilepsy, etc. From 72 h before the start of the experiment, all participants were asked to refrain from consuming alcoholic beverages, energy drinks, etc., because the consumption of these foods can change patterns related to fatigue. All participants in the experiment had at least 7 h of sleep the night before the experiment. It took about 3 months to record the physiological signals, and the signals were collected from only one participant every day between 9 and 10 AM. The reason for choosing this time period was so that all the participants were refreshed and did not feel tired.

**Figure 1:**
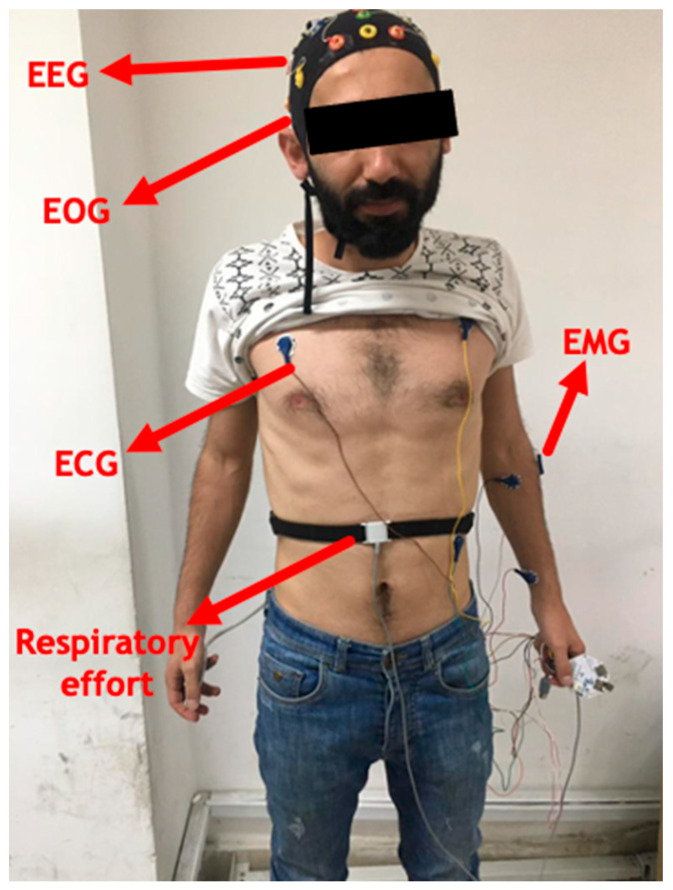
Method of connecting electrodes and leads to record EEG, EMG, ECG, EOG and respiratory effort signals in one of the participants.

This experiment was performed using 21-channel Encephalan recording devices from Medicom and Open BCI physiological signal recording module. During the experiment, EEG, ECG, EMG, and breathing rate signals were collected from the subjects while driving. An Encephalan recorder was used to record EEG signals and breathing rate, and the Open BCI module was used simultaneously to record ECG and EMG signals. The EEG signal was recorded according to the international standard 10–20 at a sampling frequency of 250 Hz. The method of obtaining these signals on the body of the participants is shown in Figure 1. Of the 21 channels related to the Encephalan device, channels A1 and A2 were selected as reference electrodes, and the remaining 19 channels were used for signal recording. Also, in order to process, according to [13,15,19], the performance of P4-C3-O1-O2 electrodes was evaluated in order to avoid increasing the computational efficiency and practicality of the current research. A Medicom sensor belt was used to record the respiratory rate signal that shows the range of participants’ breathing. As shown in Figure 1, this belt was tied around the waist of the participants above their navel and was adjusted according to the waist size of the participants. In order to record ECG signals, 3 adhesive leads were used by placing them on the chest of the participants (2 main leads and 1 reference lead around the abdomen) according to Figure 1 with a sampling frequency of 120 Hz. Three EMG sticky electrodes with a sampling frequency of 120 Hz were used on the participants’ wrists in order to detect steering wheel rotation movements as shown in Figure 1. The driving path in the simulator was regarded as a uniform highway with no traffic in order to produce mental fatigue in the driver. Additionally, automatic transmission was employed in the experiment to cause mental tiredness in the subjects. Furthermore, competitors were not permitted to surpass 100 km/h.

**Figure 2:**
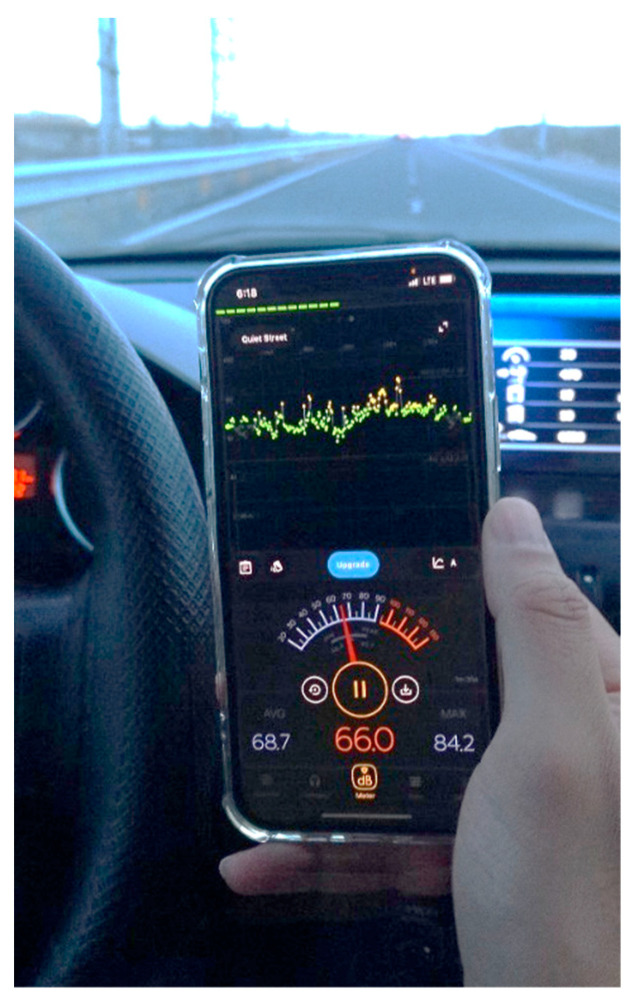
Equalization of the sound of the engine transmitted to the car cabin with the simulator environment based on the Sound Meter.

**Figure 3:**
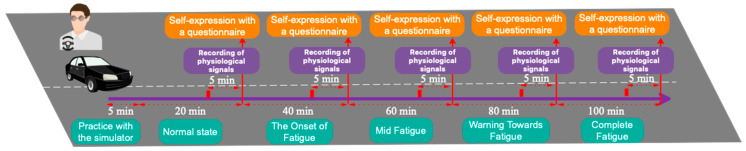
Graph depicting the fatigue levels under consideration and the signal recording procedure.

Unlike previous studies, which did not consider the sound of the engine and the surrounding environment due to the effect of noise on the recording of physiological signals and prevention of the reduction of the accuracy of their algorithm, in order to make the driving environment more realistic and guide the proposed model to the field of application, sound levels of the engine and the car cabin that were exactly in accordance with the real sample was set in the experimental environment. For this purpose, the intensity of the sound transmitted from the engine to the car cabin was extracted using the Meter Sound software while driving a standard car, and it was adjusted from the same sound intensity with the help of two stereo speakers in the simulator environment. The process of extracting sound intensity in a real driving environment in a standard car is shown in Figure 2. Before beginning the experiment, all participants drove the simulator for 5 min to get acquainted with the simulator and the goal of the experiment. Then, they continued driving for 20 min. In the last 5 min of the participants’ 20 min of driving, the recording of physiological signals started and was recorded with the label of driving in a natural state. The mentioned process was repeated 4 more times, and in the last 5 min of every 20 min period, the signal recording operation was performed and the recorded signal was labelled as fatigue onset, semi-fatigue, warning of fatigue, and full fatigue, respectively. Figure 3 shows the signal recording scenarios graphically for each of the different fatigue states. After each signal recording, the drivers reported their fatigue level using the Chalder fatigue questionnaire in order to self-evaluate their state [14]. For the first time, we investigated five alternative modes for driver fatigue, including normal, the onset of fatigue, mid fatigue, warning of fatigue, and complete fatigue, as shown in Figure 3. In Table 1, we have introduced five different modes in the form of three different scenarios in order to classify them.

**Figure 4:**
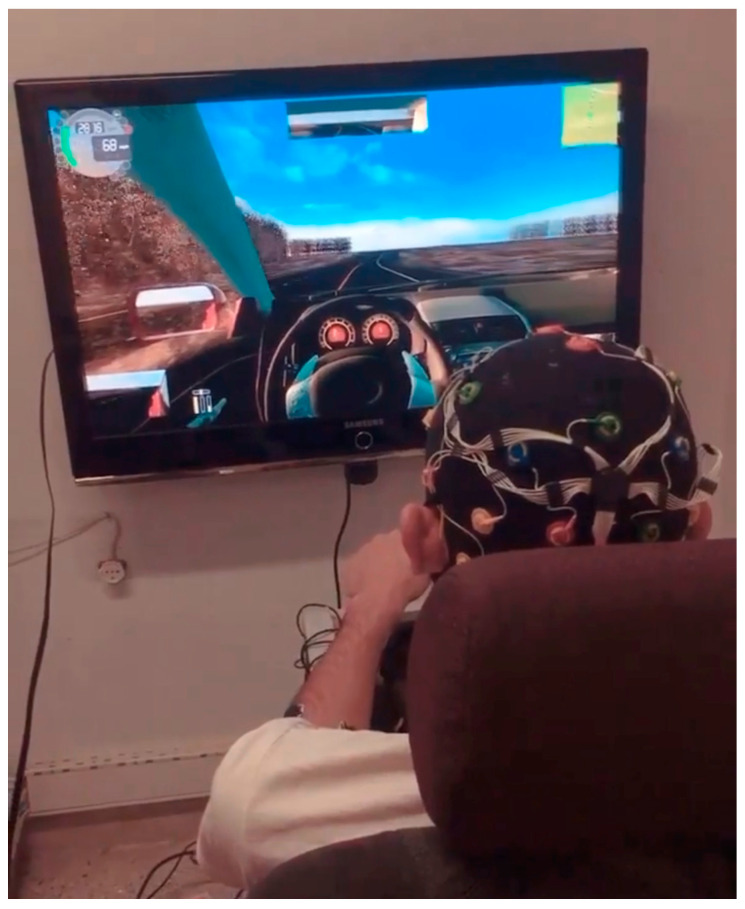
One of the participants driving in the simulator while recording physiological signals.

As discussed in Section 1, among all the physiological signals that have been used to monitor driver fatigue, EEG signals are known to the most reliable physiological signals to assess driver fatigue due to their nature. Accordingly, in the proposed registered database, the focus is on the processing of EEG signals and all processing is performed on this signal. Other recorded physiological signals, such as ECG, EMG, etc., were used only to confirm and guarantee the person’s complete fatigue visually, so that we were not just using the confirmation and guarantee of the person’s fatigue, as in previous studies, based on self-reported questionnaires by the participants during the experiment [21,22]. Figure 4 shows the driving of one of the participants in the designed simulator.

### 3.2. The Background of the Algorithms Used in this Research

In this section, the background related to the algorithms used in this study is fully reviewed.

#### 3.2.1. Generative Adversarial Network

In 2014, Goodfellow et al. [23] presented the generative adversarial network (GAN). In machine learning, GANs execute unsupervised learning tasks. These networks are made up of two models that automatically detect and learn patterns in the input data. The generator (*G*) and discriminator (*D*) are the names given to these two models. The *D* and *G* compete to evaluate, capture, and duplicate changes in the data set. GANs may be used to consistently generate new samples. The original data set can be readied for acceptance. The *G* begins by generating highly noisy signals from the input data, which is frequently delivered to the network as Gaussian noise. The G’s next task is to produce the signal as realistically as feasible in order to make the generated signal appear natural enough. The *D*, whose job is to separate the genuine signal from the false signal by inspecting the signal generated by the *G*, must determine whether the signal is natural enough or not. This process is carried out by the *G* by comparing the database signal with the signal created by the *G* [24,25]. In this network, *G* and *D* are constantly competing with each other and improving the performance of the network. To put it more simply, the *G* is responsible for producing the signal to a realistic level so that the *D* does not have the ability to distinguish it from real data. The *D* and *G* networks are trained together to maximize accuracy. During the training process of the network, the *G* tries to mislead the *D* and minimize the following equation:

(1) $$\log(1-D(G_{\left(Z\right)}))\;\begin{array}{l} \frac{\min\max}{GD}V(G,D)=E_{x}-P_{data}[\log D(x)]\\ \;+E_{pz(z)}[\log(1-D(G(Z))] \end{array}$$

The *D* in the above equation is obtained in such a way that it has the ability to distinguish real and artificial data from each other. This equation cannot be solved in a closed way and requires repetitive algorithms. The *D* has a high computational complexity, and this causes the phenomenon of overfitting to occur for the number of data with a small sample. To solve this problem, for every k optimization of the *D* function, the *G* function is optimized once [24,25].

According to the above equation, *D* is extracted in such a way that it can correctly distinguish between real and artificial data. Equation (1) cannot be solved in a closed form, so iterative and numerical methods are used to solve it. Optimizing D in the inner loop of training has a high computational complexity and will cause the phenomenon of overfitting for data with a small number of samples; therefore, for every *k* optimization of function *D*, function *G* is optimized once. For each repetition *k*, *m* number of initial noise space $p_{g}(z)$ is sampled $z=\left\{z^{\left(1\right)},\ldots ,z^{\left(m\right)}\right\}$ and *m* number of initial data distribution *P_data_* is sampled $x=\left\{z^{\left(1\right)},\ldots ,z^{\left(m\right)}\right\}$.

#### 3.2.2. Convolutional Neural Networks

Deep learning has shown to be a very useful technique due to its ability to handle huge amounts of data, and Convolutional Neural Networks (CNNs) are one of the most renowned deep neural networks. CNNs are a type of neural network that virtually perfectly replicate human vision. This is accomplished through the use of hierarchical feature extraction, which can automatically learn and extract meaningful features from images and signals for use in a variety of fields, including medicine and automated driving. Throughout the years, CNN has been a crucial component of many machine vision applications [26].

CNNs were developed and first used in the 1980s. CNN’s convolutional neural network could only distinguish handwritten numerals at the time. This network was commonly used in post offices to read postal codes, pin codes, and so on. A fundamental component of any deep learning model is that it requires a vast amount of data to train as well as a significant number of computing resources. For a long time, it was largely employed in postal departments, but with the development of powerful hardware and an increase in machine processing capacity, it was extensively embraced in a variety of disciplines, including machine vision [27].

The convolution layer serves as the foundation of any CNN working model. This is the same layer that scans pictures pixel by pixel and produces a feature map to specify future classifications. Data sampling is the name given to the pooling layer. Each feature of each convolution layer has only the information that is required. The process of building convolution layers and employing pooling is ongoing and may be repeated. Image flattener is a fully connected input layer. The outputs of the previous layer are flattened into a vector and utilized as input data for the following layer. The fully connected layer applies a random weight to the inputs and predicts an appropriate label when the feature analysis is completed and the calculation time has arrived. The fully connected output layer of the CNN model is the last layer, which retains the outputs of the labels calculated for classification and assigns a class to the images [28].

#### 3.2.3. Type-2 Fuzzy Sets

A type-2 fuzzy (T2F) set is one with fuzzy membership degrees. Such a set is useful when determining the degree of membership of a fuzzy set is challenging. A T2F system is impervious to uncertainty in fuzzy rules or system parameters [29].

As an expansion of type-I fuzzy (T1F) sets, T2F sets were introduced in 1975 [29]. The belonging functions in T2F systems feature fuzzy membership degrees as opposed to T1F systems. The use of T2F degrees of freedom has more meta parameters than T1F, that is why T2F has been widely used recently for the efficiency of control systems in neural networks [29].

Relu and Leaky Relu are two common activation functions used in deep learning networks. In the design of their architectures, researchers have only tried to change the dimensions of the number of layers and filters and have not paid attention to the main aspects such as changing the activation functions. The problem with common activation functions, including Leaky Relu, is that the value of α is always fixed and is a hyperparameter. Also, the Relu activation function is not zero-axis and returns zero as inactive on the negative axis. Using T2F units instead of these functions in convolutional networks makes the hyperparameters adjustable. Also, it solves the gradient vanishing problem and makes the algorithm converge to the desired value faster. Using these functions gives convolutional networks the ability to demonstrate better learning behavior in the network because this function is able to express linear or complex input–output maps by adjusting the uncertainty traces of its T2F sets [30].

When utilizing the T2F activation function, there are only 3C total learnable/adjustable parameters, where C is the number of hidden units, indicating that this amount is quite low when compared to the entire number of usual DNN weights. To cope with uncertainties, measurement noises, and increase detection accuracy, the appropriate functions of T2F activation functions are employed in the proposed model’s hidden layers in place of standard activation functions [30,31].

## 4. Proposed Deep Model

**Figure 5:**
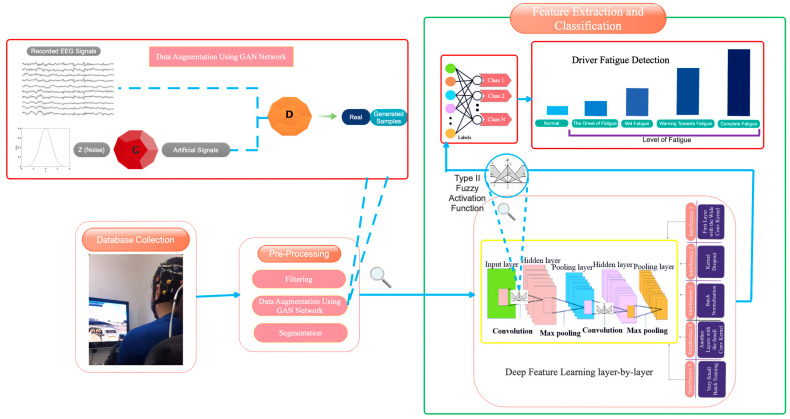
The suggested approach is depicted as a block diagram.

The proposed model for detecting five distinct levels of fatigue is provided in this section. This part covers data pre-processing, the construction of the suggested deep architecture, how to consider the training set, and deep network assessment and optimization. Figure 5 depicts the suggested model’s block diagram. Each part of the proposed block diagram will be thoroughly detailed in the sections that follow.

### 4.1. Preprocessing of Recorded Physiological Signals

Physiological signals such as EEG, EMG, ECG, and respiratory effort were acquired from participants while driving, as detailed in the signal recording section. However, only EEG signals were analyzed, and other physiological signals were used only to visually validate people’s exhaustion levels, so that the driver’s fatigue was not confirmed only based on a questionnaire and participant self-reporting, as in prior studies. The pre-processing performed on the recorded EEG data is described in full below.

To reduce the data volume and window length of recorded EEG signals, only 15 s out of 5 min of recorded signals for each fatigue level were chosen in the first step (the 15 s window length for more induction was extracted from the middle of the 5 min window length for each registered fatigue level). The 50 Hz frequency of city power was removed using a notch filter [32] in the second stage to eliminate undesired artifacts from the recorded EEG data. The data were subjected to a first-order Butterworth filter in the third phase, which ensured that only useful information in the frequency range of 0.05 to 60 Hz was included in the processing [33]. The Eye Blinking Removal plugin, which is included in the EEG Lab toolbox (ver15) [34], was used in the fourth stage to automatically eliminate the participants’ unnecessary blinks throughout the experiment. To boost detection efficiency, the data were normalized with the Min–Max normalizer [35] on a scale of 0 to 1 in the fifth phase. The GAN network was utilized in the sixth step to enhance the quantity of data points for training the proposed deep network and to avoid the phenomena of overfitting. Thus, in a GAN network, the generating network is a random vector with a uniform distribution of 100 dimensions that gives an output of 3750 × 1. The producer network’s architecture is comprised of five convolution layers with dimensions of 128, 256, 512, 1024, and 3750. Each layer of the generating network employs the Leaky Relu with a batch size of 10 and a learning rate of 0.00001. This network also has 200 repeats. To assess if the created signal is real or not, the discriminator network analyses a 3750 × one-dimensional signal. In addition, the discriminating network has five fully connected layers and two dropout layers in the first and third layers. This network employs the SGD optimizer. After utilizing the network, the number of samples grew from 3750 to 6000.

### 4.2. Deep Architecture

The suggested deep network architecture is introduced in this section. The suggested network is made up of seven convolution layers with T2F activation functions and a Softmax layer. The layers are linked as follows:

(a) A layer of dropout.
(b) A T2F activation function that includes a convolutional layer, a max pooling layer, a dropout layer, and batch normalization.
(c) The design from the previous phase is replicated six times more without the dropout layer.
(d) The Softmax layer counts points and assigns them to one of five fatigue levels, including Normal, Onset of Fatigue, Mid Fatigue, Warning Of Fatigue, and Complete Fatigue.

**Figure 6:**
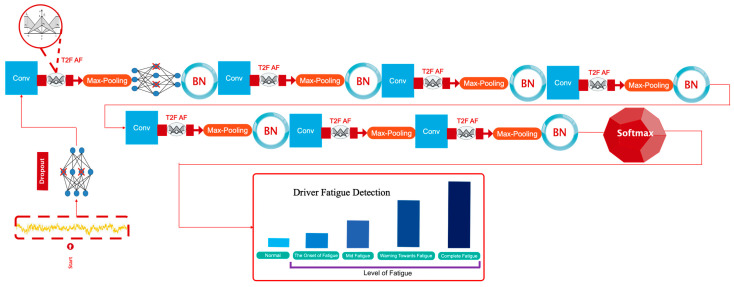
How to design the proposed architecture.

Figure 6 depicts a graphical depiction of the suggested design.

Table 2 displays the suggested architecture’s parameters, including step dimensions, layer kinds, and the number of filters. The proposed network’s weights are first assumed to be random and tiny, and they are subsequently updated using the optimal hyperparameters based on the RMSProp optimizer and the Cross-Entropy cost function described in Table 2. The suggested network design was developed utilizing various optimization techniques, several layers, and various filter sizes. Table 3 displays the recommended values for the characteristics of the proposed architecture.

### 4.3. Allocation of Training Sets, Evaluation, and How to Optimize the Proposed Deep Network

**Figure 7:**
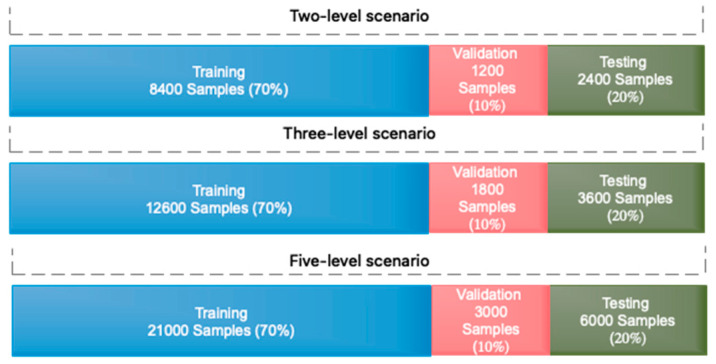
Data segmentation for training, evaluation, and validation sets in three different scenarios of driver fatigue.

The number of split samples for each of the training, validation, and assessment sets of the gathered database is specified in this section. The training set is generally composed of 70% of the data, the validation set of 10%, and the test set of 20%. The assignment of data for three different scenarios for each of the training, validation, and assessment sets is shown in Figure 7.

**Figure 8:**
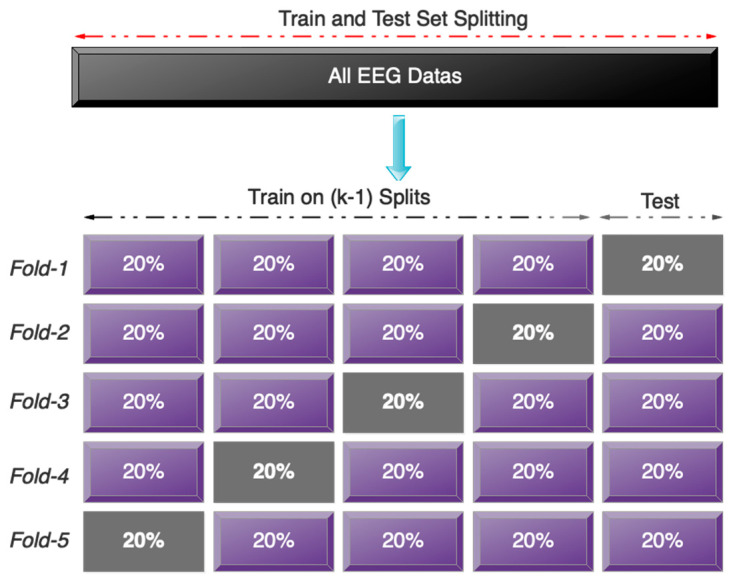
Graphic display for performing five-fold cross-validation.

In addition to the random data allocation described above, we used five-fold cross-validation for the collected data set to ensure that all of the collected data were used in the training and testing process and that the phenomenon of over-fitting did not occur in the training process. Figure 8 graphically depicts the five-fold cross-validation on the recorded data.

## 5. Results

This section contains the subsections listed below. The simulation results are provided first. The current study is compared to earlier studies one by one in the following section. The latest approaches are then simulated using the collected database and compared to the proposed network. Finally, in the discussion section below, we show how to confirm weariness based on visual observations.

All pre-processing was conducted in MATLAB 2021 software, and the suggested network was designed in Python platform using the Google Club Premium Plus edition.

### 5.1. The Results Obtained from the Proposed Deep Network

**Figure 9:**
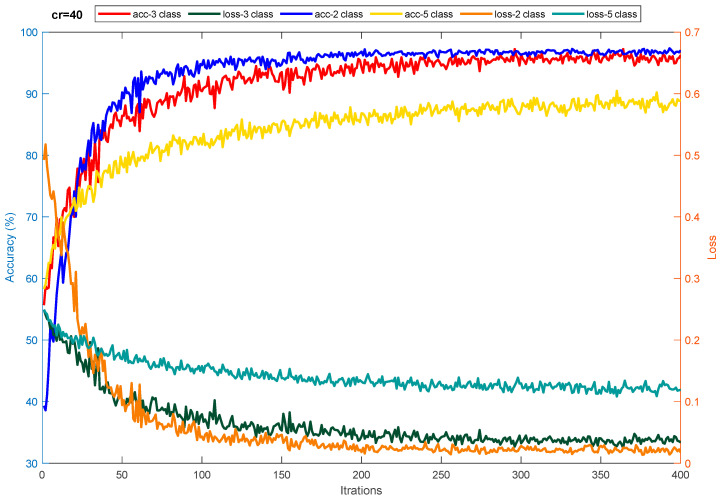
Performance of the proposed model in terms of error criteria and network accuracy after 400 repetitions in detecting three distinct situations.

**Figure 10:**
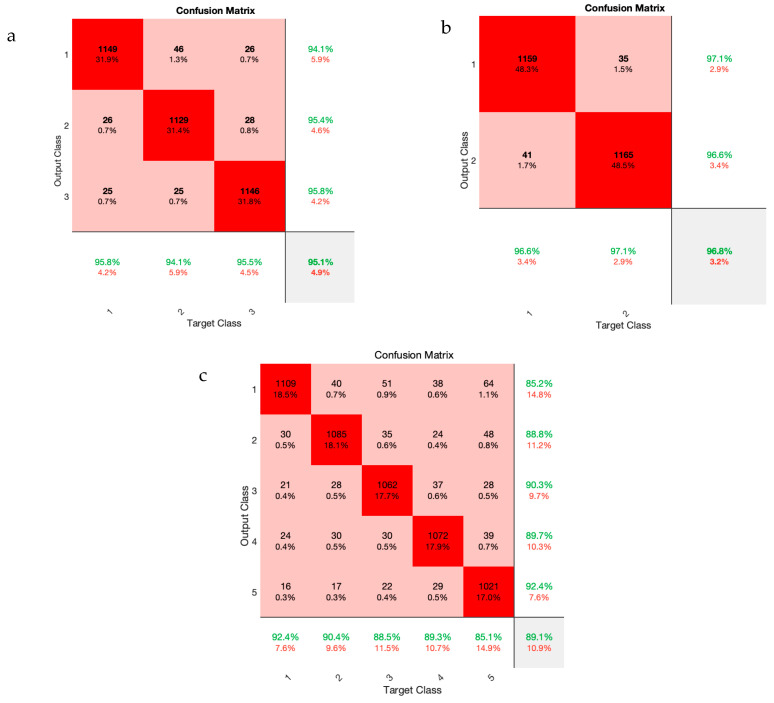
Confusion matrix related to 3 different scenarios (**a**–**c**) of driver fatigue.

**Figure 11:**
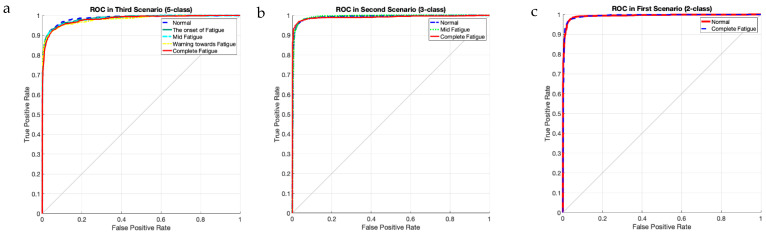
ROC analysis related to 3 different scenarios (**a**–**c**) of driver fatigue.

**Figure 12:**
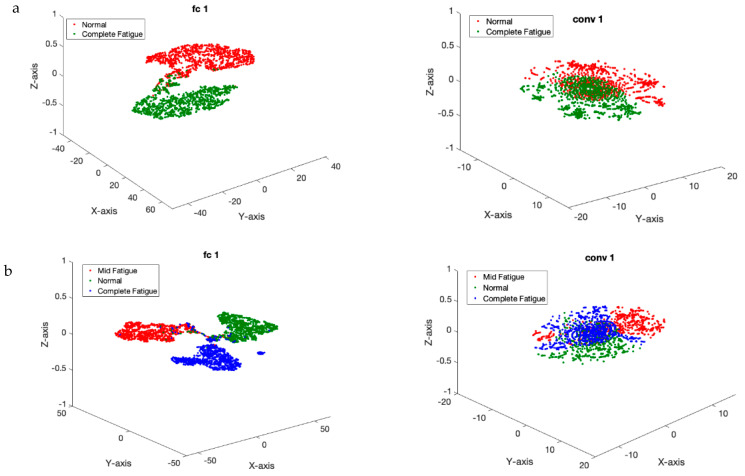

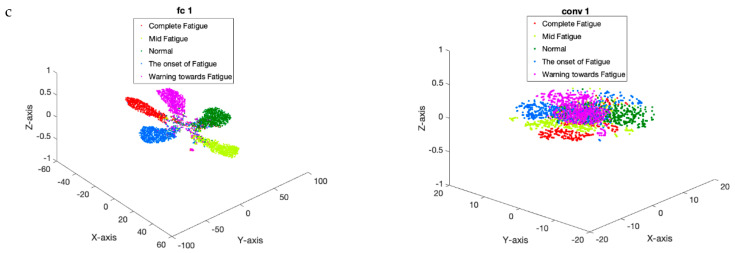
t-SNE analysis considered for two input and output layers in 3 fatigue scenarios (**a**–**c**).

**Figure 13:**
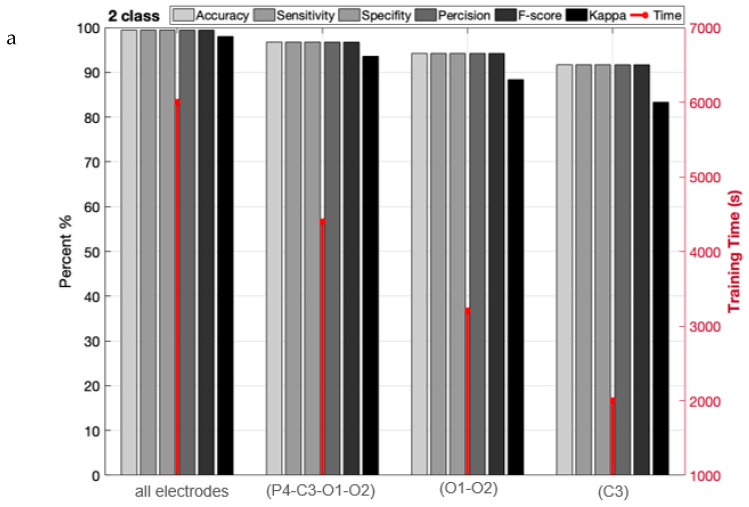

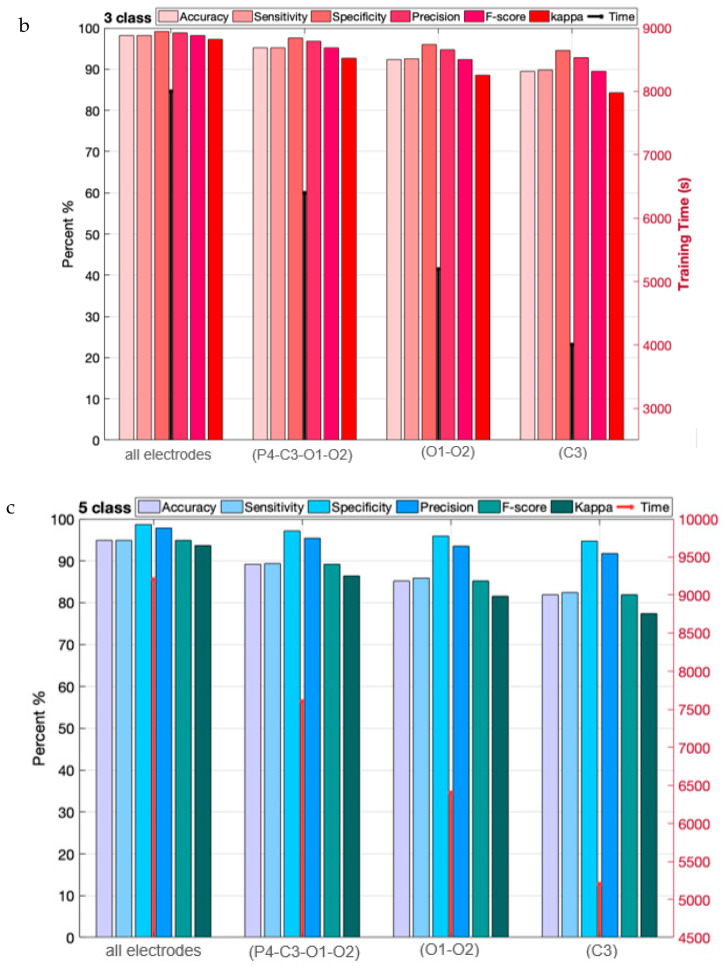
The suggested model’s performance in terms of accuracy, precision, sensitivity, F-score, kappa, and training duration for three different scenarios (**a**–**c**).

**Figure 14:**
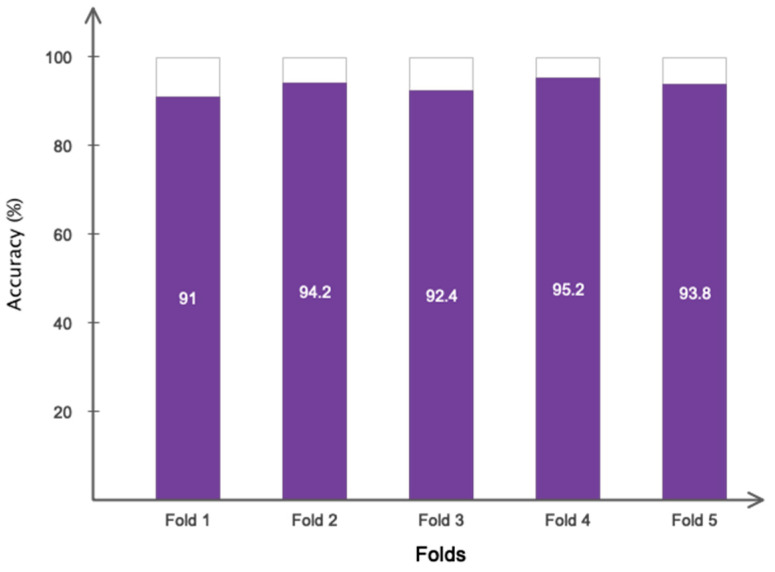
The classification accuracy for the third scenario based on 5-fold cross-validation.

**Figure 15:**
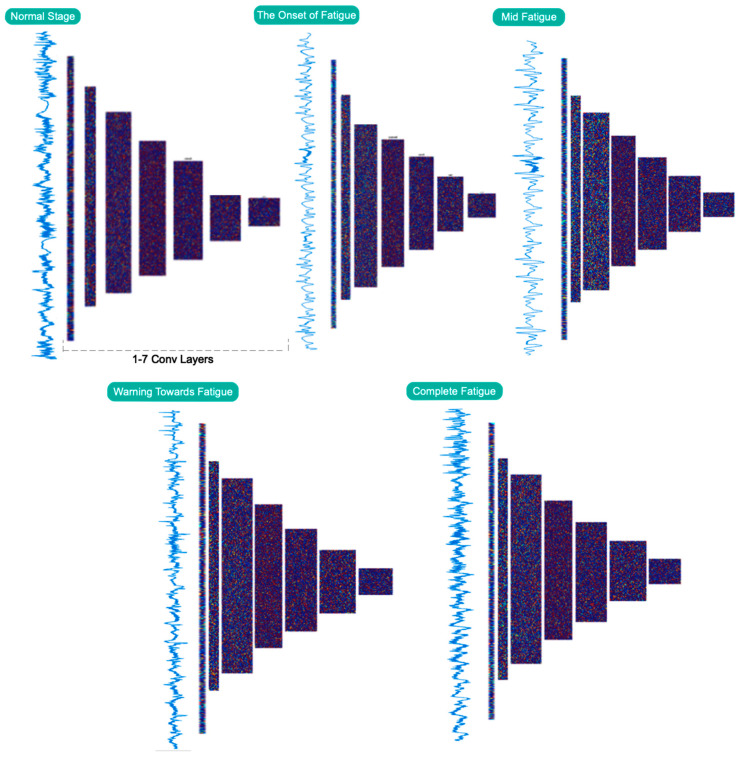
Each network layer is visually analyzed for five different tiredness stages.

Table 1 is used to assess the simulation results for the proposed multi-level database in this section. This table presents two three-level and five-level situations for the first time in a previous study. As a result of the multi-class situations, a dependable method for monitoring driver fatigue may be offered. Table 4 displays the accuracy gained using the suggested model for each scenario for chosen electrodes and all electrodes. As can be observed from the table, the attained accuracy in the five-level scenario is greater than 89%. According to Table 4, it is obvious that the usage of 19 electrodes outperforms the selected electrodes. Except for one instance, the accuracy attained for the selected electrodes is greater than 90%. As is obvious, using all of the electrodes to detect driver fatigue in practical applications is impractical and impossible. Accordingly, the rest of the presented results are based on the selected electrodes. Figure 9 depicts the network’s accuracy and error for all two-level, three-level, and five-level situations investigated using the suggested model. According to this figure, the network attained a stable state in roughly 180 iterations for the two-level scenario. Furthermore, for three-level and five-level situations, the network was stable after around 300 and 450 iterations, respectively. Furthermore, the acquired accuracy for all cases was greater than 85%, according to this figure. According to the same figure, the network error based on the suggested network for two-level, three-level, and five-level scenarios decreased from 0.5, 0.23, and 0.25 to roughly 0.01, 0.003, and 0.13, respectively. The confusion matrix for two-level, three-level, and five-level situations is shown in Figure 10. As observed in the two-level case, only 41 samples out of 1165 were incorrectly recognized and categorized. Figure 11 depicts the ROC curve for two-level, three-level, and five-level situations. As can be seen in the image, all of the values obtained on the left half of the curve are in the range of 0.85 to 1, indicating that the suggested model performs optimally. Figure 12 displays the t-SNE graph for the Conv_1 and FC_1 layers in the two-, three-, and five-level situations. Figure 12 illustrates that the proposed network is efficient in distinguishing class samples from each other and has a high accuracy in separating inter-class data from each other. According to this chart, for the well-known two-level scenario, practically all samples of normal condition and full exhaustion are segregated from one another. Figure 13 depicts the suggested model’s performance in terms of several assessment parameters such as sensitivity, accuracy specificity, precision, kappa, F-score, and training duration for all electrodes and chosen electrodes for various situations. As a result, as can be shown, lowering the number of electrodes reduces the network’s training time. Furthermore, as is obvious, the suggested model’s performance in scenarios built using various assessment criteria is highly promising. As stated in the data allocation section, five-fold cross-validation was used on the collected data in this study to ensure that the phenomenon of overfitting did not occur during the training process. Figure 14 depicts the results of the five-fold cross-validation for all electrodes in the third scenario. As is well known, the accuracy obtained for each fold was greater than 90%, and thus the phenomenon of overfitting did not occur. Figure 15 depicts the visualization of the different levels of the proposed network in order to better properly portray network performance for the five-level scenario. According to this figure, as shown by the visualization of different layers, the proposed network was able to extract different features from each class, which is consistent with the qualitative and visual evaluation results for fatigue confirmation (reviewed in the discussion section).

### 5.2. How to Confirm Fatigue Based on Physiological Signals

**Figure 16:**
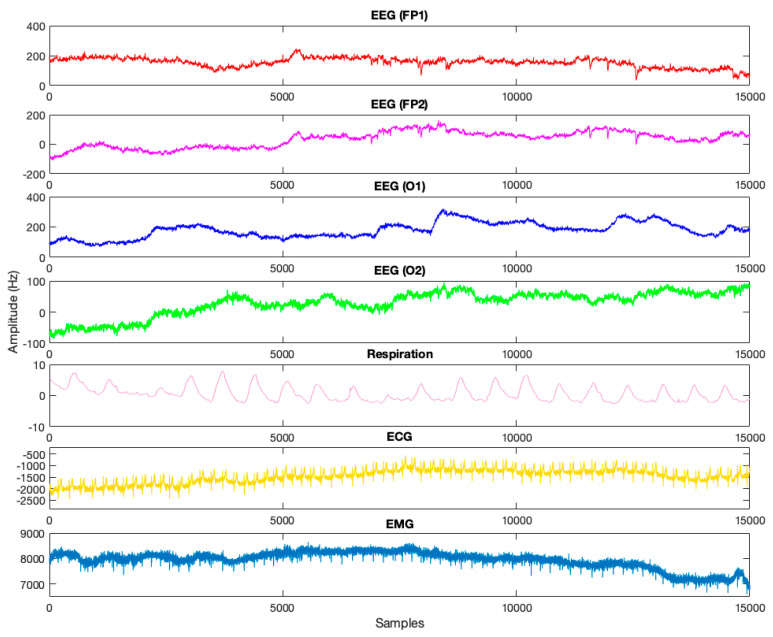

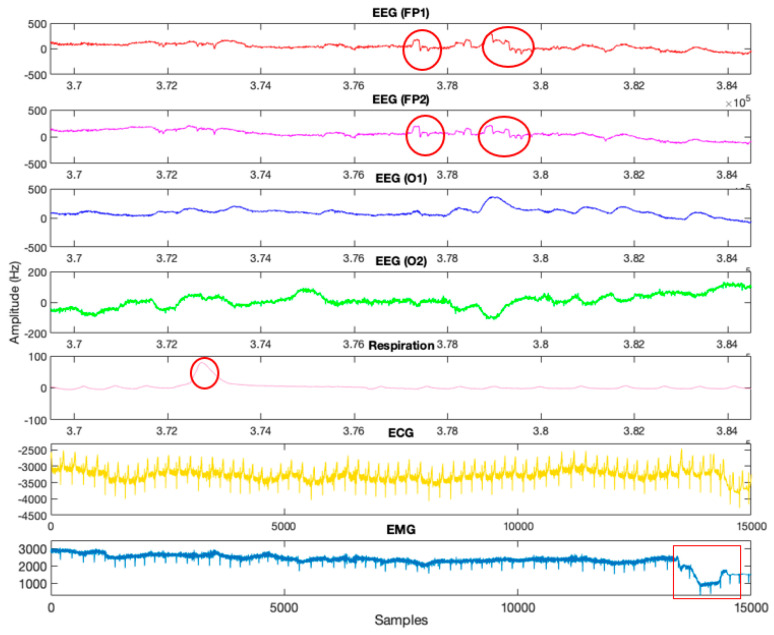
Physiological analysis to confirm fatigue in one of the test participants.

Figure 16 shows the physiological signals for the two states of normal and full fatigue during a 1 min recording for one participant. According to what was said, in this work, in addition to confirming the fatigue based on the drivers’ self-reporting, qualitative analysis of physiological signals by a neuroscientist has been used to ensure the person’s fatigue. According to Figure 16, for the channels Fp1 and Fp2, which are the manifestation of the participant’s blinks, as can be seen, the number of participant’s blinks decreased in the state of complete fatigue. Even in the section marked in the figure, as is clear, the participant is in a fully closed eyelid state (sleep–wake state) during part of the experiment. For the O1 and O2 electrodes of the EEG signal, which are related to the occipital regions, in the state of complete fatigue, compared to the normal state, the amplitude of the signal decreases and shifts from alpha to theta waves. In addition, the breaths of the participant in the state of full fatigue change from the state of full breaths to the state of clipping, according to the respiration signal. Also, based on the ECG signal, if the QRS peaks are counted for the normal state and full fatigue, it is clear that the number of heartbeats in the full-fatigue state is significantly reduced. According to the EMG signal, the movement impulses of the steering wheel by the participant in the fatigue state decrease compared to the normal state, which cause a decrease in the signal amplitude. Also, a car accident with a guardrail also occurred, as indicated in the section indicated in the picture, in the case of a driver in a state of complete fatigue. As mentioned in previous sections, all previous fatigue studies have only used questionnaires to confirm driver fatigue, which is inaccurate and can cause bias on the part of drivers. As a result, physiological examination, unlike the previous studies that examine only two states, can help to consider more levels of fatigue. However, physiological examination can be annoying for drivers due to the placement of multiple electrodes to record EMG signals.

### 5.3. Comparison with Recent Studies

The automatic identification of driver fatigue on two levels has been the topic of much study in recent years. Table 5 compares the best findings given in these studies to the suggested model. As shown in the chart, earlier research employed manual feature extraction approaches to identify driver weariness, which might be troublesome in real-time systems. Other approaches based on feature learning, on the other hand, have shown low accuracy. As demonstrated in Table 5, the suggested model based on feature learning has the best performance when compared to prior research.

As shown in Table 1, the accuracy attained for the classification of two classes has nearly reached human accuracy (above 95%) in our investigation and all recent studies. However, no recent research has reported on the impact of engine and cabin noise because taking into account the test conditions described in Section 3.1 will affect categorization accuracy. Furthermore, none of the recent studies included multi-mode fatigue in their research, and the conclusions were based on a binary classification. This verifies the suggested model’s best performance for autonomous driver tiredness identification.

### 5.4. Using Recent Methods and Algorithms to Compare with the Proposed Model

A one-to-one comparison with earlier studies is illogical because all earlier databases used various databases, which were frequently private. Furthermore, unlike the databases utilized in earlier studies, the suggested database has five levels, and studies based on several levels should be established in the future for comparison. To assess the ideal performance of the suggested technique, we simulated existing algorithms utilized in recent research to automatically identify driver fatigue using the proposed multi-level collected database and compared them to our own proposed algorithm. In this respect, two feature learning approaches based on raw signal and manual feature extraction were utilized, as well as standard and popular algorithms and classifications such as MLP [36], SVM [37], CNN [38] with Relu activation function [39], CNN with Leaky Relu activation function [40], Deep Boltzmann Machines (DBMs) [41], and the suggested model for the five-level scenario. The mean, peak coefficient, skewness, variance, maximum, minimum, and kurtosis were retrieved from the recorded EEG signals for engineering characteristics. The suggested network design in Figure 6 was explored for CNN without the use of a T2F activation function. The SVM kernel was polynomial, and the network search method was utilized to optimize kernel parameters. In addition, for MLP and DBM networks, the number of hidden layers and learning rate were set to 3 and 0.001, respectively. Table 6 shows the accuracies acquired using feature learning from EEG and manual features. As demonstrated in the table, the application of feature learning in deep networks has resulted in a considerable improvement in accuracy. As can be shown, the suggested network outperforms other deep and traditional techniques in terms of accuracy when compared to the manual feature extraction strategy.

**Figure 17:**
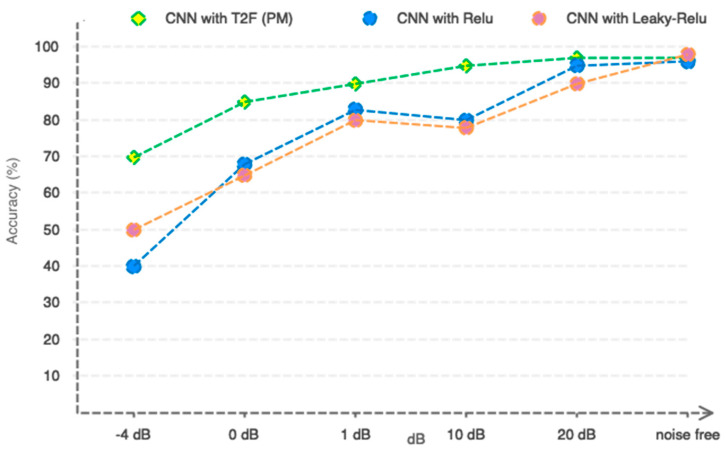
The suggested model’s resistance curve to external noise.

As shown in Table 5, the usage of CNN with Relu, Leaky Relu, and T2F activation functions performs nearly identically. However, in order to explain why we chose T2F activation functions over functions like Relu and Leaky Relu, we decided to introduce white Gaussian noise to the EEG signals and measure the resistance of our approach at various SNRs. Figure 17 depicts the performance of three distinct activation functions. As is commonly known, all three functions operate almost identically in noise-free mode. However, when noise levels rise, the two activation mechanisms of Relu and Leaky Relu are insufficient to defend. The T2F activation function, on the other hand, has been shown to be resistant to a wide spectrum of SNRs. As a result, when SNR = 90, the two-class classification accuracy remains over 90%. The explanation for the suggested network’s optimum resistance may be due to the following reasons:

- T2F activation functions are used instead of Relu and Leaky Relu activation functions.
- The initial convolutional layer has high filter dimensions, whereas the subsequent convolutional layers have lower filter dimensions.

Despite the positive performance of the suggested strategy, this study, like prior investigations, contains flaws. The following are examples of this study’s shortcomings:

1. The EEG data were recorded using wet electrodes, which might be problematic in real-life situations; as a result, the effectiveness of dry electrodes should also be assessed.
2. Due to cost constraints, the camera was not employed in this study to capture the participants’ facial expressions and test events while they were driving. To more properly measure and validate the participants’ weariness, the performance of the camera used to record the events during the test must be evaluated and investigated.
3. The circumstances addressed for signal recording in this study are solely connected to passive tiredness; however, in real scenarios, exhaustion generated by stress and fatigue produced by effort should be explored as well.

## 6. Conclusions

Using recorded EEG data, this work proposes an automatic technique for multi-level detection of driver fatigue. This study applied deep learning methodologies such as GAN and CNN. The suggested network design in this study consists of seven convolutional layers with T2F activation functions, and a Softmax layer to allocate points to each class. According to this research, the suggested approach has a high accuracy of 89% in classifying five stages of driver fatigue. Furthermore, the proposed architecture offers high resistance to external disturbances across a broad range of SNRs. Considering the high resistance of the proposed architecture in noisy environments, the current model can be used with high reliability in real-time driver fatigue detection systems. Despite the favorable performance of the present study, wet electrodes were used in this research, and it is necessary to examine the performance of dry electrodes in future studies. Also, in future studies, it is necessary to evaluate the performance of pre-trained networks to reduce the algorithm’s computational load for real-time applications. Due to the ideal effectiveness of the proposed algorithm, the present model can be considered by automobile companies to build smart cars.

## Figures and Tables

**Table 1:** Scenarios and modes considered for driver fatigue.

Scenarios	Modes
**Two-level**	Normal vs. Complete Fatigue
**Three-level**	Normal vs. Mid Fatigue vs. Complete Fatigue
**Five-level**	Normal vs. Onset of Fatigue vs. Mid Fatigue vs. Warning Of Fatigue vs. Complete Fatigue

**Table 2:** Details about the proposed architecture’s dimensions of filters, steps, and so on.

L	Layer Type	Activation Function	Output Shape	Size of Kernel and Pooling	Strides	Number of Filters	Padding
1	ConV 1-D	T2F	(None, 2375, 16)	128 × 1	20 × 1	16	**yes**
2	Max-Pool 1-D	-	(None, 1187, 16)	2 × 1	2 × 1	-	**no**
3	ConV 1-D	T2F	(None, 1187, 32)	3 × 1	1 × 1	32	**yes**
4	Max-Pool 1-D	-	(None, 593, 32)	2 × 1	2 × 1	-	**no**
5	ConV-D	T2F	(None, 593, 64)	3 × 1	1 × 1	64	**yes**
6	Max-Pool 1-D	-	(None, 296, 64)	2 × 1	2 × 1	-	**no**
7	ConV 1-D	T2F	(None, 296, 64)	3 × 1	1 × 1	64	**yes**
8	Max-Pool 1-D	-	(None, 148 64)	2 × 1	2 × 1	-	**no**
9	ConV1-D	T2F	(None, 148, 64)	3 × 1	1 × 1	64	**yes**
10	Max-Pool1-D	-	(None, 74, 64)	2 × 1	2 × 1	-	**no**
11	ConV1-D	T2F	(None, 74, 64)	3 × 1	1 × 1	64	**yes**
12	Max-Pool 1-D	-	(None, 37, 64)	2 × 1	2 × 1	-	**no**
13	ConV1-D	T2F	(None, 37, 64)	3 × 1	1 × 1	64	**yes**
14	Max-Pool 1-D	-	(None, 18, 64)	2 × 1	2 × 1	-	**no**
15	Softmax	-	(None, 2-3-5)	-	-	-	

**Table 3:** The optimal parameters in the suggested model.

**Parameters**	**Search space**	**Optimal Value**
Optimizer	RMSProp, Adam, Sgd, Adamax, Adadelta	Adadelta
Cost function	MSE, Cross-entropy	Cross-Entropy
Number of Convolution layers	3, 5, 7, 9, 11	7
Number of Filters in the first convolution layer	16, 32, 64, 128	16
Number of Filters in the second convolution layer	16, 32, 64, 128	32
Number of Filters in another convolution layers	16, 32, 64, 128	64
Size of filter in the first convolution layer	3, 16, 32, 64, 128	128
Size of filter in another convolution layers	3, 16, 32, 64, 128	3
Dropout rate before the first convolution layer	0, 0.2, 0.3, 0.4, 0.5	0.3
Dropout rate after the first convolution layer	0, 0.2, 0.3, 0.4, 0.5	0.3
Batch size	4, 8, 10, 16, 32, 64	10
Learning rate	0.01, 0.001, 0.0001	0.001

**Table 4:** In three cases, the classification accuracy results of chosen electrodes and all electrodes are considered.

	19 Channels			4 Electrodes (P4-C3-O1-O2)		
	2 Levels	3 Levels	5 Levels	2 Levels	3 Levels	5 Levels
**ACC (%)**	99.50	98.13	94.89	96.83	95.11	89.15

**Table 5:** An assessment of the performance of the proposed model in contrast to that of the existing literature.

**References**	**Method**	**Accuracy (%)**
*Luo* et al. [12]	Adaptive Multiphase Entropy	95
*Sheykhivand* et al. [13]	CNN-LSTM	99
*Zeng* et al. [14]	EMD-SVM	94
*Sheykhivand* et al. [15]	CS-CNN	96.36
*Subasi* et al. [16]	Flexible WT-SVM	97
*Wang* et al. [17]	GLU	87
*Zheng* et al. [18]	EMD-RF	97
*Chen* et al. [19]	RMS-U-Net	95
*Shahbakhti* et al. [20]	WT-Adaboost	90
**Our model**	GAN-CNN with T2F	**2-level Scenario = 96.8** **3-level scenario = 95.1** **5-level Scenario = 89.1**

**Table 6:** A comparison of the suggested model’s performance with those of current research.

Model	Feature Learning (%)	Eng Features (%)
**MLP**	72.6	84.4
**DBM**	87.3	74.6
**SVM**	89.8	76.4
**CNN with Relu**	96.1	90
**CNN with Leaky-Relu**	96.4	90.5
**Our Model (CNN with T2F)**	96.8	91.2

## Data Availability

Tabriz University’s ethics committee in Tabriz, Iran. Data access is private and not publicly available.
